# Aneurisma isolado de artéria femoral superficial roto contido: relato de caso

**DOI:** 10.1590/1677-5449.007517

**Published:** 2017

**Authors:** Daiane Cristina Ferreira Damasceno, Júlio Beserra Evaristo, Geraldo Felipe, André Luiz Guimarães Câmara, Cláudio Eluan Kalume, Alcides José Araújo Ribeiro, Leonardo Pires de Sá Nóbrega

**Affiliations:** 1 Hospital de Base do Distrito Federal – HBDF, Unidade de Cirurgia Vascular e Angiologia – UCIVASA, Brasília, DF, Brasil.

**Keywords:** artéria femoral superficial, aneurisma roto, cirurgia

## Abstract

Aneurismas verdadeiros isolados da artéria femoral superficial (AFS) são eventos raros. Manifestam-se principalmente em homens idosos e frequentemente estão associados a outros aneurismas. Possuem etiologia variada e costumam ser detectados quando apresentam complicações como trombose, embolização distal ou, mais raramente, ruptura. O presente caso refere-se a um paciente cujo aneurisma de AFS se apresentou roto contido e sem associações com outros aneurismas. Foram realizados eco-Doppler colorido arterial, que diagnosticou a ruptura, e angiotomografia, que evidenciou aneurisma sacular de AFS medindo 11,4 × 8,8 cm, com grande trombo mural. Uma arteriografia foi utilizada para programação de revascularização, e detectou-se leito distal via artéria tibial anterior. O paciente foi submetido a revascularização cirúrgica convencional eletiva em artéria femoropoplítea distal com veia safena ipsilateral invertida, com sucesso. Apresentou como complicação pós-operatória infecção de sítio cirúrgico. A pesquisa microbiológica teve resultado negativo, e o estudo anatomopatológico confirmou aneurisma verdadeiro da AFS.

## INTRODUÇÃO

Aneurismas verdadeiros isolados da artéria femoral superficial (AFS) são eventos raros^1^ e representam 0,5% dos aneurismas periféricos^2^ e 1% de todos os aneurismas de artéria femoral^3^. Acometem principalmente homens idosos^1^^,^ 4 ^,^ 5 e o membro direito^5^ e costumam estar associados a outros aneurismas (aortoilíacos, femorais ou poplíteos)^1^^,^ 6. A etiologia desses aneurismas pode ser aterosclerótica, micótica, por infecção por HIV, autoimune ou pode estar relacionada à síndrome de Marfan^7^.

O trajeto anatômico da AFS, envolto por grandes massas musculares na coxa, dificulta a detecção precoce dos aneurismas e, muitas vezes, o primeiro sintoma é a ruptura^8^. Em contrapartida, nos aneurismas da artéria femoral comum e poplítea, a ruptura é incomum^5^. Quando há ruptura, o quadro clínico pode envolver isquemia distal, massa pulsátil na coxa ou massa pulsátil e dolorosa^4^.

As principais complicações dos aneurismas da AFS são trombose, embolização distal ou, mais raramente, ruptura^1^^,^ 2 ^,^ 4 ^,^ 5. Devido à alta incidência de complicações, a ressecção e a revascularização devem ser realizadas. Um rastreamento à procura de outros aneurismas arteriais em outras localizações é mandatório^4^.

O presente relato de caso apresenta um aneurisma de AFS roto em um paciente idoso no qual o tratamento cirúrgico convencional foi realizado.

## RELATO DE CASO

Paciente masculino, 74 anos, pardo, ex-tabagista (interrompera o hábito havia 17 anos), portador de hipertensão arterial e doença pulmonar obstrutiva crônica (DPOC). Não apresentava história pregressa de cirurgias e traumas. Deu entrada na unidade de emergência com queixa de dor leve associada a abaulamento na face anteromedial da coxa esquerda e edema em todo o membro, iniciados havia 30 dias.

**Figura 1:**
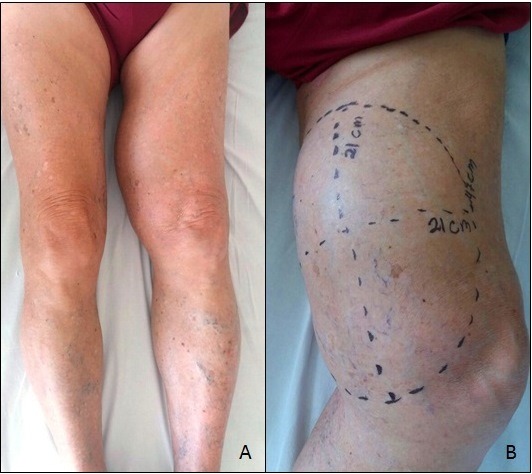
Pré-operatório: (A) vista anterior, com aumento do volume da coxa esquerda; (B) vista medial, com dermografia dos diâmetros externos do aneurisma.

Ao exame físico, apresentava-se corado e hemodinamicamente estável. Ausculta cardíaca e pulmonar se mostrou fisiológica. Exame abdominal estava normal. Na avaliação do membro inferior direito, todos os pulsos arteriais estavam presentes e, no membro inferior esquerdo, evidenciava-se grande abaulamento na coxa, medindo 21 × 21 cm em seus maiores diâmetros, endurecido, sem frêmito ou pulso (Figura 1). O paciente apresentava pulsos femoral e poplíteo palpáveis e normais e pulsos distais ausentes.

**Figura 2:**
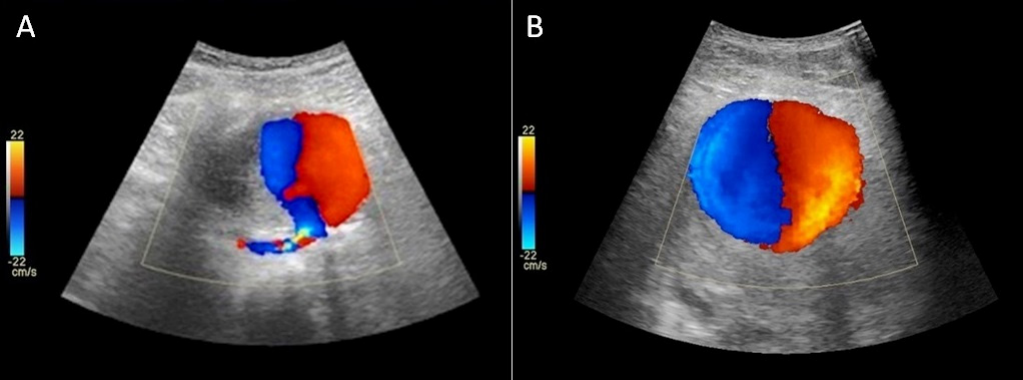
Eco-Doppler colorido arterial: (A) imagem longitudinal do aneurisma sacular em conexão com a artéria femoral superficial esquerda; (B) sinal do *yin-yang* indicando fluxo bidirecional dentro do aneurisma.

Foram realizados exames de laboratório que não identificaram alterações em hemograma, eletrólitos, função renal ou coagulograma. Na avaliação inicial, foi realizado eco-Doppler colorido arterial, que identificou volumoso aneurisma sacular no terço médio e distal da AFS esquerda, com dissecção e ruptura de suas paredes, e sua luz media 5,8 × 4,7 cm nos maiores diâmetros (Figura 2). Além disso, havia fluxo bidirecional no interior da imagem sacular e na comunicação com a AFS (Figura 2). No eco-Doppler colorido venoso, notou-se a presença de imagem ecogênica na luz da veia poplítea, com perda total de sua compressibilidade, caracterizando um quadro de trombose venosa profunda (TVP).

Como o paciente estava hemodinamicamente estável e apresentava quadro de DPOC sem acompanhamento regular, além da não identificação do leito distal nos exames iniciais, optou-se por uma investigação mais detalhada do caso para posterior programação cirúrgica eletiva. Dessa forma, foi iniciada, ainda na unidade de emergência, a anticoagulação terapêutica devido ao quadro de TVP em veia poplítea esquerda.

**Figura 3:**
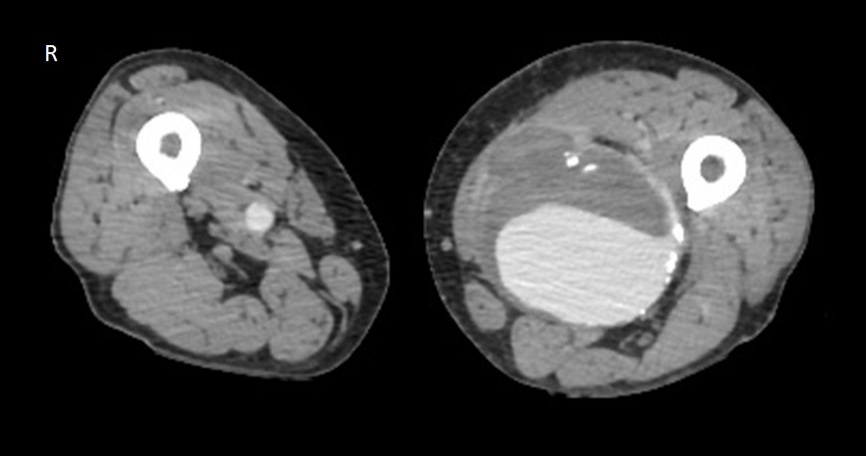
Angiotomografia computadorizada evidenciando dilatação aneurismática sacular no terço distal de artéria femoral superficial esquerda, medindo 11,4 × 8,8 cm em seus maiores eixos transversais, com grande trombo mural.

Na avaliação complementar, foi solicitada uma angiotomografia computadorizada de membros inferiores (Figura 3), que identificou dilatação aneurismática sacular no terço distal da AFS esquerda, medindo 11,4 × 8,8 cm em seus maiores eixos transversais, com grande trombo mural e luz remanescente de 6,6 cm no maior eixo visibilizado.

**Figura 4:**
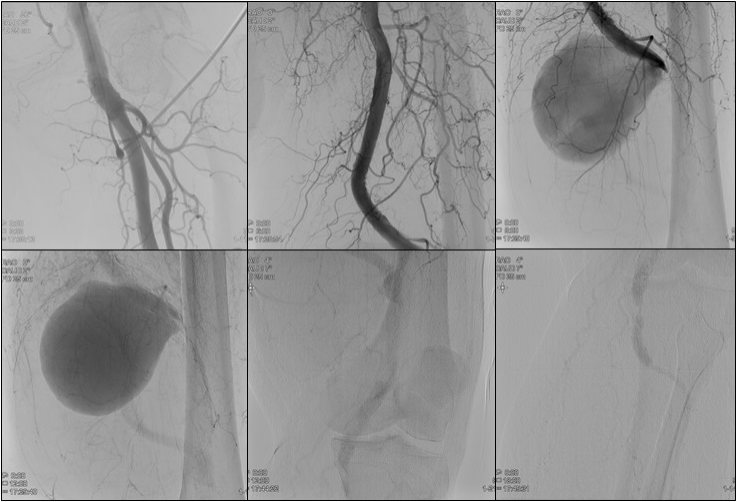
Arteriografia mostrando aneurisma sacular no terço distal da artéria femoral superficial com oclusão da artéria poplítea. Escassa circulação colateral. Reenchimento da artéria poplítea abaixo do aneurisma e *outflow* via artéria tibial anterior.

O paciente foi submetido a investigação de outros aneurismas com eco-Doppler colorido de aorta, artérias ilíacas e artérias do membro inferior direito, sem detecção de aneurismas nesses sítios. Para programação cirúrgica, foi realizada uma arteriografia de membro inferior esquerdo (Figura 4), que mostrou a presença de grande aneurisma no terço distal da AFS esquerda, com colo curto, oclusão da artéria poplítea e reenchimento desta abaixo do aneurisma pela escassa circulação colateral e deságue através da artéria tibial anterior.

**Figura 5:**
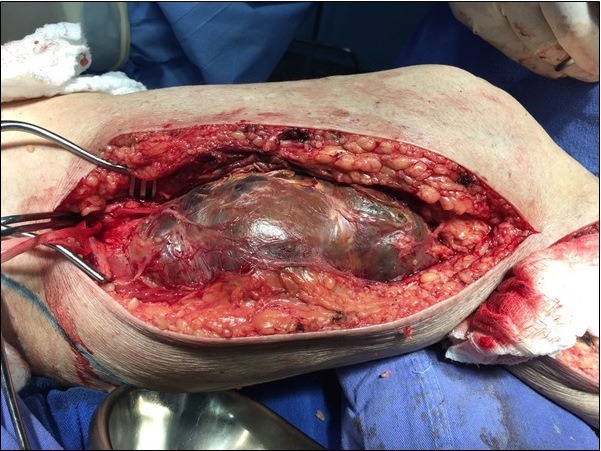
Intraoperatório: dissecção do saco aneurismático.

O paciente foi submetido a cirurgia eletiva com incisão na face medial da coxa esquerda para controle proximal e distal ao aneurisma. Foi identificado volumoso hematoma encapsulado na coxa esquerda (Figura 5), o que confirmou o diagnóstico de aneurisma roto contido de AFS. Foram realizadas aneurismectomia e reconstrução do segmento vascular com veia safena magna esquerda invertida. Foram enviados fragmentos de tecidos ressecados para histopatologia e microbiologia. Não houve crescimento de microrganismos, e o resultado da biópsia do tecido foi aneurisma verdadeiro de AFS esquerda.

O paciente apresentou boa evolução no pós-operatório: membro inferior esquerdo aquecido, perfusão periférica < 3 s, presença de pulsos femoral, poplíteo, tibial anterior e pedioso palpáveis e normais, ausência de hematomas e sinais de infecção no sítio cirúrgico. Recebeu alta hospitalar no quinto dia de pós-operatório em boas condições clínicas e em uso de rivaroxabana, a ser seguido por um período de seis meses para tratamento de TVP de veia poplítea esquerda.

No 15º dia de pós-operatório, o paciente retornou à unidade de emergência devido a hiperemia e saída de secreção em sítio cirúrgico. Foi, então, internado para realização de antibioticoterapia venosa por um período de 10 dias.

**Figura 6:**
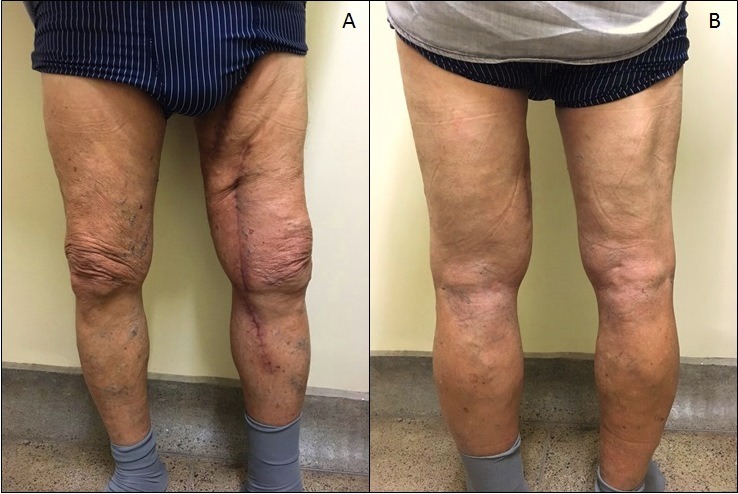
Seguimento pós-operatório de 3 meses: (A) vista anterior e (B) vista posterior.

No retorno ambulatorial, aos três meses de seguimento (Figura 6), apresentava boa cicatrização do sítio cirúrgico e regressão do edema no membro inferior. O eco-Doppler colorido arterial de controle de membro inferior esquerdo evidenciou enxerto pérvio, sem sinais de trombose ou estenose no enxerto e nas anastomoses.

## DISCUSSÃO

Aneurismas ateroscleróticos verdadeiros isolados da AFS são raros^1^. Essa doença ocorre mais na população idosa e mais frequentemente em homens que em mulheres (3:1)^9^. São bilaterais em 18% dos casos^6^ e frequentemente associados a aneurismas em outras localizações (27-69% dos casos)^10^.

Os aneurismas periféricos das artérias dos membros são geralmente palpados facilmente, mas os aneurismas da AFS são de difícil detecção precoce^8^, pois a sua porção medial e distal fica abaixo da fáscia muscular e entre os músculos sartório, adutor longo e vasto medial. Dessa forma, não são diagnosticados até que ocorram complicações^7^.

A incidência de associação com aneurismas aortoilíacos, aneurismas de artéria poplítea e outros aneurismas periféricos é, respectivamente, 40-69%, 54% e 27%^11^. Assim, uma vez diagnosticado um aneurisma de AFS, é mandatória uma investigação de aneurismas arteriais concomitantes em outras localizações^4^.

Aneurismas de artérias periféricas podem estar associados a vários fatores etiológicos, como sífilis, desordens imunológicas (doença de Behçet), inflamatórias (granulomatose de Wegener), do tecido conjuntivo (síndrome de Ehlers-Danlos ou de Marfan), ou ainda a fatores secundários (fibrodisplasia ou malignidade)^7^^,^ 11 ^-^ 14. Na ausência de fatores etiológicos claros, grande parte dos aneurismas são classificados como ateroscleróticos, até mesmo quando existe pouca ou nenhuma evidência de aterosclerose em outros vasos^9^.

As complicações estão presentes em 65% dos casos de aneurismas de AFS, segundo Rigdon e Monajjem^9^, incluindo ruptura (35%), trombose (18%) e eventos embólicos distais (12%). Essas complicações ocorrem com menos frequência na comparação com pacientes com aneurisma de artéria poplítea^3^. Assim, aneurismas sintomáticos, com diâmetro superior a 2,5 cm ou mais de duas vezes o calibre normal da artéria, devem ser reparados para prevenir complicações que ameacem a viabilidade do membro^15^^,^ 16.

O diagnóstico precoce desses aneurismas de AFS é de suma importância para evitar as complicações no membro, além de permitir um planejamento cirúrgico eletivo com menor morbimortalidade ao paciente. Eco-Doppler colorido arterial, angiotomografia e angiorressonância são úteis no diagnóstico, na avaliação das relações anatômicas do aneurisma com as estruturas adjacentes e também no planejamento cirúrgico. A arteriografia fica reservada para a investigação do leito distal para revascularização, se necessário.

O tratamento cirúrgico convencional de aneurisma periférico permanece como padrão-ouro^16^^,^ 17, com a utilização de anastomose terminoterminal, enxerto venoso (preferencialmente com veia autóloga) ou prótese^3^. Nos casos eletivos, observa-se perviedade de aproximadamente 80% em dois anos para enxerto venoso, comparado a 65% para politetrafluoretileno^3^. Outras técnicas podem ser utilizadas, como a simples ligadura do aneurisma sem a revascularização arterial posterior, desde que o paciente com arteriopatia conhecida apresente boa circulação colateral, que permita a viabilidade e preservação da extremidade^18^. Técnicas endovasculares com colocação de *stents* recobertos podem ser utilizadas enm pacientes com alto risco cirúrgico (idosos com mais de 70 anos), mas a patência desses enxertos em longo prazo ainda não é definida^19^.

As principais complicações pós-operatórias são: infecção de sítio cirúrgico, infecção protética, trombose precoce ou tardia do enxerto, estenose do enxerto, falso aneurisma anastomótico e linfedema^20^. O seguimento pós-operatório varia de acordo com o conduto utilizado na revascularização arterial. Quando é usado o conduto venoso, o eco-Doppler colorido arterial é realizado 30 dias após o procedimento; trimestralmente, durante 1 ano após a primeira avaliação; semestralmente, durante os 2 anos seguintes; e anualmente a partir dessa data. No caso de conduto protético, o seguimento deve ser decidido caso a caso, pois não há evidência científica provando o custo-benefício dessa vigilância^20^.

Diante do exposto, conclui-se que o aneurisma de AFS é um evento raro e sua principal apresentação é a ruptura. Pode estar associado a outros aneurismas, e o rastreamento de aneurismas em outras localizações é mandatório. O tratamento cirúrgico convencional ainda é a melhor opção devido à durabilidade do enxerto, mas apresenta os riscos inerentes à própria cirurgia. O seguimento do paciente deve acontecer de modo a realizar a vigilância do enxerto e evitar as complicações tardias do pós-operatório.
