# Supplementary material for: Aneurisma isolado de artéria femoral superficial roto contido: relato de caso
Source: J Vasc Bras. 2017 Oct-Dec;16(4):348–54. [Article in Portuguese] doi: 10.1590/1677-5449.007517 (PMC5944316; doi:10.1590/1677-5449.007517)
Supplement: Translation

# Translation: Isolated ruptured aneurysm of the superficial femoral artery: case report

**Authors:** Daiane Cristina Ferreira Damasceno, Júlio Beserra Evaristo, Geraldo Felipe, André Luiz Guimarães Câmara, Cláudio Eluan Kalume, Alcides José Araújo Ribeiro, Leonardo Pires de Sá Nóbrega

**Affiliations:** 1 Hospital de Base do Distrito Federal – HBDF, Unidade de Cirurgia Vascular e Angiologia – UCIVASA, Brasília, DF, Brazil.

**Keywords:** superficial femoral artery, ruptured aneurysm, surgery

## Abstract

Isolated true aneurysms of the superficial femoral artery (SFA) are rare events. They mostly manifest in elderly men and are frequently seen in conjunction with other aneurysms. They have varied etiology and are usually detected when they complicate with thrombosis or distal embolization, or, more rarely, when they rupture. The present case report describes a patient with an aneurysm of the SFA that was ruptured and contained and who had no other aneurysms. Color Doppler ultrasound of the arteries revealed the rupture and angiotomography showed a saccular aneurysm of the SFA measuring 11.4 × 8.8 cm, with a large mural thrombus. Arteriography was used to plan revascularization and showed the distal bed with outflow via the anterior tibial artery. The patient was treated with conventional elective distal femoropopliteal surgical revascularization with the ipsilateral saphenous vein inverted, which was successful. Recovery was complicated by a postoperative surgical site infection. Microbiology tests were negative and the anatomopathological study confirmed a true aneurysm of the SFA.

## INTRODUCTION

Isolated true aneurysms of the superficial femoral artery (SFA) are rare events,^1^ accounting for 0.5% of peripheral aneurysms^2^ and 1% of all femoral artery aneurysms.^3^ They are primarily found in elderly men^1^^,^ 4 ^,^ 5 and in the right limb^5^ and tend to be seen in conjunction with other aneurysms (aortoiliac, femoral, or popliteal).^1^^,^ 6 The etiology of these aneurysms may be atherosclerotic, mycotic, secondary to HIV infection, autoimmune, or related to Marfan Syndrome.^7^

The anatomic course of the SFA through the thigh is surrounded by large muscle masses, making early detection of aneurysms less likely, and the first symptom is very often rupture.^8^ In contrast, common femoral or popliteal artery aneurysms rarely rupture.^5^ When rupture does occur, clinical presentation may include distal ischemia, a pulsating mass in the thigh or a pulsating and painful mass.^4^

The major complications of SFA aneurysms are thrombosis, distal embolization or, more rarely, rupture.^1^^,^ 2 ^,^ 4 ^,^ 5 Resection and revascularization should be performed because of the high incidence of complications. It is also obligatory to screen for additional arterial aneurysms in other locations.^4^

This case report describes a ruptured SFA aneurysm in an elderly patient who was treated with conventional surgery.

## CASE REPORT

The patient was a 74-year-old, brown-skinned, male, ex-smoker (he had quit 17 years previously), with arterial hypertension and chronic obstructive pulmonary disease (COPD). He had no prior history of surgery or traumas. He presented at the emergency room complaining of mild and swelling on the anteromedial surface of his left thigh and edema of the entire limb, with onset 30 days previously.

At the physical examination he had good color and was hemodynamically stable. Cardiac and pulmonary auscultation were normal. Abdominal examination was unremarkable. Examination of the right lower limb found all arterial pulses were present and examination of the left lower limb revealed considerable swelling of the thigh, reaching 21 × 21 cm at its largest diameters, hardened and without thrill or pulse (Figure 1). The patient had palpable and normal femoral and popliteal pulses, but distal pulses were absent.

**Figure 1:** Preoperative: (A) anterior view, with increased volume of the left thigh; (B) medial view, with external diameters of the aneurysm marked on the skin.

Laboratory tests were ordered, but hemogram, electrolytes, renal function, and coagulogram were all normal. Investigation was initiated with color Doppler ultrasound of the arteries, which showed a voluminous saccular aneurysm in the mid and distal thirds of the left SFA, with dissection and rupture of the walls and a lumen measuring 5.8 × 4.7 cm at its largest diameters (Figure 2). There was also bidirectional flow inside the sac and through its communication with the SFA (Figure 2). Color Doppler ultrasound of the veins showed an echogenic image in the lumen of the popliteal vein, which had entirely lost its compressibility, characteristic of deep venous thrombosis (DVT).

**Figure 2:** Color Doppler ultrasound of the arteries: (A) longitudinal image of the saccular aneurysm communicating with the left superficial femoral artery; (B) *Yin-yang* sign indicating bidirectional flow within the aneurysm.

Since the patient was hemodynamically stable and had COPD but was not in regular follow-up, and also because the distal bed had not been identified in the workup examinations, the decision was taken to conduct a more detailed investigation of the case and plan elective surgery for a later date. Therapeutic anticoagulation was initiated in the emergency unit, because of the DVT in the left popliteal vein.

As part of the supplementary investigation, computed tomography angiography of the lower limbs was conducted (Figure 3), showing a saccular aneurysmal dilatation in the distal third of the left SFA, measuring 11.4 × 8.8 cm at its largest transverse axes, with a large mural thrombus, and the remaining lumen measuring 6.6 cm at the largest visible axis.

**Figure 3:** Computed tomography angiography showing saccular aneurysmal dilatation of the distal third of the left superficial femoral artery, measuring 11.4 x 8.8 cm at its largest transverse axes, with large mural thrombus.

The patient was examined with color Doppler ultrasound to screen for other aneurysms in the aorta, iliac arteries, and arteries of the right lower limb, but no aneurysms were detected in any of these areas. Arteriography of the left lower limb was conducted to aid in planning surgery (Figure 4), showing a large aneurysm of the distal third of the left SFA, with a short neck and occlusion of the popliteal artery, which was refilled below the aneurysm by scant collateral circulation and outflowed to the anterior tibialis artery.

**Figure 4:** Arteriography showing saccular aneurysm of the distal third of the superficial femoral artery with occlusion of the popliteal artery. Scant collateral circulation. Refilling of the popliteal artery below the aneurysm and outflow via the anterior tibialis artery.

The patient underwent elective surgery, with an incision in the medial surface of the left thigh for proximal and distal control of the aneurysm. A voluminous encapsulated hematoma was found in the left thigh (Figure 5), confirming the diagnosis of contained ruptured aneurysm of the SFA. Aneurysmectomy was performed, followed by reconstruction of the vascular segment with the left great saphenous vein inverted. Fragments of dried tissues were sent for histopathology and microbiology. No microorganisms grew and the tissue biopsy result was true aneurysm of the left SFA.

**Figure 5:** Intraoperative image: dissection of the aneurysm sac.

The patient recovered well during the postoperative period: the left lower limb was warm, with peripheral perfusion < 3 s, femoral, popliteal, anterior tibialis and pedal pulses were all present, palpable, and normal, and there were no hematoma or signs of infection of the surgical site. The patient was discharged from hospital on the fifth day after the operation in good clinical condition, with a 6-month prescription for Rivaroxaban to treat the left popliteal vein DVT.

Fifteen days after the operation the patient returned to the emergency unit because of hyperemia and secretions from the surgical site. He was admitted to hospital for venous antibiotic treatment lasting 10 days.

At the 3-month clinical follow-up consultation (Figure 6), the surgical site was healing well and the edema of the lower limb was in regression. Color Doppler ultrasound of the arteries of the left lower limb showed that the graft was patent, with no signs of thrombosis or stenosis of the graft or at the anastomoses.

**Figure 6:** Three-month follow-up: (A) front view and (B) rear view.

## DISCUSSION

Isolated true atherosclerotic aneurysms of the SFA are rare.^1^ This condition occurs in the elderly population and affects men more often than women (3:1).^9^ In 18% of case they are bilateral^6^ and they are often seen in conjunction with aneurysms in other sites (27-69% of cases).^10^

Peripheral aneurysms of arteries in the limbs are generally easily palpated, but aneurysms of the SFA are unlikely to be detected early,^8^ because their medial and distal portions lie below the fascia musculature and between the sartorius, adductor longus, and vastus medialis muscles. They therefore remain undiagnosed until they cause complications.^7^

The incidence of rates of concomitant aortoiliac aneurysms, popliteal artery aneurysms, and other peripheral aneurysms are, respectively, 40-69%, 54%, and 27%.^11^ Therefore, once an SFA aneurysm has been diagnosed, investigation to screen for additional arterial aneurysms in other sites is obligatory.^4^

Aneurysms of peripheral arteries can be linked to a variety of etiologic factors, such as syphilis, immunological disorders (Behçet’s disease), inflammatory conditions (Wegener’s granulomatosis), connective tissue diseases (Ehlers-Danlos or Marfan Syndrome), or even to secondary factors (fibrodysplasia or malignancy).^7^^,^ 11 ^-^ 14 In the absence of clear etiologic factors, a large proportion of aneurysms are classified as atherosclerotic, even when there is little or no evidence of atherosclerosis in other vessels.^9^

According to Rigdon and Monajjem^9^, complications are seen in 65% of cases of SFA aneurysm and include rupture (35%), thrombosis (18%), and distal embolic events (12%). These complications occur with less frequency in comparison with patients with popliteal artery aneurysms.^3^ Nevertheless, symptomatic aneurysms, with diameters greater than 2.5 cm or more than twice the normal caliber of the artery should be repaired to prevent complications that threaten the viability of the limb.^15^^,^ 16

Early diagnosis of SFA aneurysms is extremely important to avoid complications affecting the limb, in addition to allow for planning of elective surgery, with lower morbidity and mortality. Color Doppler ultrasound of the arteries, angiotomography, and magnetic resonance angiography are useful for diagnosis, for assessing anatomic relationships between the aneurysm and adjacent structures, and for planning surgery. Arteriography can be reserved for assessing the distal bed for revascularization, if necessary.

Conventional surgical treatment remains the gold standard for peripheral aneurysms,^16^^,^ 17 with end-to-end anastomosis, venous grafting (preferably with an autologous vein), or prosthetic grafts.^3^ In elective cases, patency rates with vein grafts are approximately 80% at 2 years, compared with 65% for polytetrafluoroethylene.^3^ Other techniques can be used, such as simple ligature of the aneurysm without later arterial revascularization, as long as patients with known arteriopathy have good collateral circulation, permitting viability and preservation of the extremity.^18^ Endovascular techniques with placement of covered stents can be used for patients with high surgical risk (the elderly over the age of 70 years), but the long term patency of these grafts is unknown.^19^

The principal postoperative complications are infection of the surgical wound, prosthetic infection, early or late thrombosis of the graft, stenosis of the graft, false anastomotic aneurysm and lymphedema.^20^ Postoperative follow-up varies depending on the methods of arterial revascularization employed. When a venous graft is constructed, color Doppler ultrasound is used to examine the arteries 30 days after the procedure; three-monthly for 1 year after the first assessment; six-monthly for the following 2 years; and annually thereafter. If a prosthetic graft has been used, follow-up should be decided on a case-by-case basis, because there is no scientific evidence demonstrating the cost-benefit relationship of such surveillance.^20^

In view of the above, it is concluded that an SFA aneurysm is a rare event and that it most often presents as a rupture. It may be seen in combination with other aneurysms and screening for aneurysms of other sites is obligatory. Conventional surgical treatment is still the best option because of the durability of grafts, but involves the risks inherent to surgery. Patients should be followed-up to monitor the graft and avoid late postoperative complications.
